# Challenges in Diagnosing Intermediate Maple Syrup Urine Disease by Newborn Screening and Functional Validation of Genomic Results Imperative for Reproductive Family Planning

**DOI:** 10.3390/ijns7020025

**Published:** 2021-05-14

**Authors:** Mona Sajeev, Sharon Chin, Gladys Ho, Bruce Bennetts, Bindu Parayil Sankaran, Bea Gutierrez, Beena Devanapalli, Adviye Ayper Tolun, Veronica Wiley, Janice Fletcher, Maria Fuller, Shanti Balasubramaniam

**Affiliations:** 1 Genetic Metabolic Disorders Service, Western Sydney Genetics Program, The Children’s Hospital at Westmead, Sydney, NSW 2145, Australia; mona.sajeev@health.nsw.gov.au (M.S.); bindu.parayilsankaran@health.nsw.gov.au (B.P.S.); 2 Genetics and Molecular Pathology, SA Pathology at Women’s and Children’s Hospital, North Adelaide, SA 5006, Australia; sharon.chin@sa.gov.au (S.C.); janice.fletcher@health.nsw.gov.au (J.F.); Maria.fuller@adelaide.edu.au (M.F.); 3 Department of Molecular Genetics, Western Sydney Genetics Program, The Children’s Hospital at Westmead, Sydney, NSW 2145, Australia; gladys.ho@health.nsw.gov.au (G.H.); bruce.bennetts@health.nsw.gov.au (B.B.); 4 Discipline of Genetic Medicine, Sydney Medical School, The University of Sydney, Sydney, NSW 2006, Australia; adviye.tolun@health.nsw.gov.au (A.A.T.); veronica.wiley@health.nsw.gov.au (V.W.); 5 The Children’s Hospital at Westmead Clinical School, Faculty of Medicine & Health, Sydney Medical School, The University of Sydney, Sydney, NSW 2006, Australia; 6 NSW Biochemical Genetics Service, Western Sydney Genetics Program, The Children’s Hospital at Westmead, Sydney, NSW 2145, Australia; Bea.Gutierrez@health.nsw.gov.au (B.G.); beena.devanapalli@health.nsw.gov.au (B.D.); 7 NSW Newborn Screening Programme, Western Sydney Genetics Program, The Children’s Hospital at Westmead, Sydney, NSW 2145, Australia; 8 Adelaide Medical School, University of Adelaide, Adelaide, SA 5005, Australia

**Keywords:** intermediate MSUD, VOUS, *DBT* gene, leucine decarboxylation studies, validation of pathogenicity

## Abstract

Maple syrup urine disease is caused by a deficiency of branched-chain alpha-ketoacid dehydrogenase, responsible for degradation of leucine, isoleucine, and valine. Biallelic pathogenic variants in *BCKDHA*, *BCKDHB*, or *DBT* genes result in enzyme deficiency. We report the case of a female infant who presented with mild gross motor delay at 4 months, and seizures with hypoglycaemia at 5 months. Newborn screening returned total leucine/isoleucine at the 99.5th centile of the population; however, as second-tier testing reported minimal alloisoleucine, the results were considered inconsistent with MSUD. Plasma amino acid and urine organic acid analyses at 5 months were, however, consistent with a diagnosis of MSUD. A brain MRI showed bilateral symmetrical T2 hyperintense signal abnormalities involving white matter, globus pallidus, thalamus, brainstem, and dentate nuclei with restricted diffusion. A repeat MRI 10 months post-dietary-intervention showed the resolution of these changes and progression in myelination. Her clinical phenotype, including protein tolerance, correlated with intermediate MSUD. Molecular analysis of all three genes identified two variants of uncertain significance, c.434-15_434-4del and c.365A>G (p. Tyr122Cys) in the *DBT* gene. The rate of leucine decarboxylation in fibroblasts was reduced, but not to the extent observed in classical MSUD patients, supporting an intermediate form of MSUD. Previously reported mRNA splicing studies supported a deleterious effect of the c.434-15_434-4del variant. This functional evidence and confirmation that the variants were in trans, permitted their reclassification as pathogenic and likely pathogenic, respectively, facilitating subsequent prenatal testing. This report highlights the challenges in identifying intermediate MSUD by newborn screening, reinforcing the importance of functional studies to confirm variant pathogenicity in this era of molecular diagnostics.

## 1. Synopsis

The identification of biallelic VUS in a 5-month-old female infant with intermediate MSUD necessitated leucine decarboxylation in fibroblasts, for orthogonal validation of the identified molecular variants.

## 2. Introduction

Maple syrup urine disease (MSUD, OMIM #248600) is an autosomal recessive disorder caused by decreased activity of branched-chain alpha-ketoacid dehydrogenase (BCKD) complex, a mitochondrial enzyme involved in the degradation of branched-chain amino acids (leucine, isoleucine, valine) and their ketoacid derivatives, with resultant generation of alloisoleucine, the pathognomonic disease marker [1]. A pan-ethnic disorder affecting approximately 1:185,000 live births [1], it can be as high as 1 in 176 live births in old-order Mennonites in Pennsylvania [2].

BCKD is composed of three catalytic components including: E1, a decarboxylase with two subunits, E1α- and E1β, that require thiamine pyrophosphate as a coenzyme; E2, dihydrolipoyl acyltransferase; and E3, dihydrolipoamide dehydrogenase [3]. MSUD is caused by biallelic pathogenic variants in any one of the three genes that encode the BCKD subunits, *BCKDHA* (MSUD type 1A)*, BCKDHB* (type 1B), and *DBT* (type II), which encode the E1 α, E1 β, and E2 subunits respectively [1]. Digenic inheritances of a *BCKDHA* and a *DBT* gene variant have been reported in three related patients with intermittent phenotype, partially responsive to thiamine [4]. As the E3 subunit is also involved in the pyruvate dehydrogenase and alpha-ketoglutarate dehydrogenase complexes, MSUD ‘type 3’ presents with a markedly different phenotype with congenital lactic acidosis, branched-chain alpha-ketoaciduria, and alpha-ketoglutaric aciduria [3].

Accumulations of leucine and alpha-ketoisocaproic acid (aKIC) cause metabolic intoxication, characterised by developmental delay, encephalopathy, anorexia, feeding problems, and movement disorders [1]. The brain is particularly vulnerable to the inhibitory effect of leucine as it has a high affinity (low Km) for the common L-type amino acid transporter 1 (LAT1) which transports nine essential amino acids across the blood–brain barrier. Secondary neurotransmitter (dopamine and serotonin, derived from tyrosine and tryptophan) deficiency, attributable to the unbalanced transport of essential amino acids, leads to movement disorder manifestations [5]. Additionally, disruption of energy metabolism secondary to branched-chain ketoacid accumulation reportedly underlies the neuropathology in MSUD mouse models [6]. In comparison, increased isoleucine or valine manifest little apparent toxicity [3].

The clinical phenotype of MSUD varies across a spectrum depending on residual activity of the BCKD complex and is independent of the affected gene [1]. The most severe form is ‘classical MSUD’, (0–2% residual enzyme activity), which commonly presents with neonatal-onset encephalopathy and premature death if untreated, and accounts for approximately 75–80% of affected infants [5]. The mildest form is ‘intermittent MSUD’ with >40% residual activity and is only symptomatic when acute decompensation occurs, often triggered by catabolic states such as infection or stress [5]. There appears to be no good genotype –phenotype correlation [7]. Prior to these episodes, children grow and develop normally; however, decompensation is significant and can lead to developmental delay, progressive encephalopathy, and premature death if left untreated. ‘Intermediate MSUD’ refers to phenotypes in between the two aforementioned subtypes [1]. Both intermittent and intermediate MSUD may not be identified by newborn screening, due to partial branched-chain alpha-ketoacid dehydrogenase deficiency with concomitant normal BCAA. ‘Thiamine-responsive’ MSUD is another reported phenotype, which presents similarly to intermediate MSUD and demonstrates partial responsiveness to thiamine [5]. However, it is not known with certainty if individuals with true thiamine-responsive MSUD exist [1].

Tandem mass spectrometry (MS/MS) expanded newborn screening (NBS) can identify elevations of the sum total BCAAs on dried blood samples (DBS), without distinguishing the individual isobaric amino acids (leucine, isoleucine, and alloisoleucine) [8]. Determination of each branched-chain amino acid requires incorporation of a second tier MS/MS assay using column separation. A positive NBS result requires follow-up biochemical testing with quantitative plasma amino acid including alloisoleucine analyses. The presence of plasma alloisoleucine, above 5 μmol/L is the most sensitive and specific diagnostic marker for all forms of MSUD, with concentrations exceeding this value reported in 94% and 99.9% of patients with intermediate and classic forms of MSUD, respectively [9]. Elevated BCAAs and alloisoleucine on quantitative plasma amino acid profiling provides diagnostic confirmation of MSUD [1]. Urine organic acid analysis by GC-MS/MS detects BCKA (including alpha-ketoisocaproate, alpha-keto- beta-methylisovalerate, and alpha-ketoisovalerate), providing diagnostic support for MSUD. Genetic confirmation is afforded by identification of biallelic pathogenic variants in *BCKDHA, BCKDHB*, or *DBT* [1]. Enzymatic studies on cultured fibroblasts, leukocytes, or biopsied liver tissue have also been used for diagnostic confirmation [1]. It has been suggested that in vitro measurements of BCKD activity are discordant with in vivo leucine oxidation rates [9] or dietary leucine tolerance, hence are not clinically useful [1]. However, these results from leucine decarboxylation studies in fibroblasts concur with the clinical phenotype of intermediate MSUD, hence indicating the potential usefulness of such studies in classifying clinical phenotypes.

## 3. Clinical Summary

The proband initially presented at 5 months of age following a generalised tonic-clonic seizure (5–10 min) associated with hypoglycaemia (BSL 2.2 mmol/L). There was no preceding illness and the hypoglycaemia resolved promptly with an oral feed. She was reviewed by a neurologist who initiated a metabolic work-up. The past history was notable for motor developmental delay with poor head control and a mild episode of bronchiolitis at 4 months of age. There was a family history of hypothyroidism and right-sided aortic arch variant in the mother and epilepsy in a maternal uncle.

She is the first-born child of non-consanguineous Caucasian parents, with an unremarkable birth history, being born at 38 weeks via Caesarean section for breech position, with APGARs 9 and 9, birth weight of 3430 g (50–85th centile), and head circumference of 36 cm (85–97th centile). The pregnancy and postnatal course were uneventful. A routine underivatised NBS sample collected at 59 h of life, on breastfeeding, showed slightly increased “total leucines” (362 μmol/L whole blood, in-house cut-off 99.5th centile of the population <350), which was followed up by a second-tier DBS testing to distinguish individual BCAAs. Leucine and isoleucine were moderately increased in this analysis (271 μmol/L, RI: 35–154; 91 μmol/L, RI: 12–80, respectively), and valine was not analyzed. At the time, overall results were interpreted as not being consistent with MSUD. A retrospective review at the time of clinical presentation of the analytical tracing determined alloisoleucine was slightly elevated at 5.8 μmol/L whole blood (RI: <5).

Upon presentation at 7 months, there was a history of regression of gross motor milestones, and delayed speech and language development over the preceding week. She displayed truncal hypotonia, brisk deep tendon reflexes with downgoing plantars, and abnormal jerky limb movements. Cardiovascular, respiratory, and abdominal examinations were normal, and no dysmorphic features were identified. Laboratory parameters included mild respiratory alkalosis with pH 7.47 (RI 7.34–7.43), pCO_2_ 27 mm Hg (RI 32–45), pO_2_ 72 mm Hg (RI 80–100), HCO_3_ 19.1 mmol/L (RI 18.0–24.0), base excess −3.2 mmol/L (RI −4.0–3.0); normal sodium 137 mmol/L (RI 133–144), potassium 4.4 mmol/L (RI 3.5–5.4), chloride 103 mmol/L (RI 97–110), urea 2.9 mmol/L (RI 1.0–6.0), creatinine 18 μmol/L (RI 11–36); and calculated serum osmolarity 280 mmol/kg (RI 275–295). Urine organic acid analysis detected 2-hydroxy, 3-methylvalerate, and 2-hydroxyisovalerate–markers of MSUD. Plasma amino acid analysis confirmed elevated BCAA leucine 2614 μmol/L (RI 56–178 μmol/L), isoleucine 885 μmol/L (RI 34–106 μmol/L), valine 1044 μmol/L (RI 108–314 μmol/L), and alloisoleucine 291 μmol/L (RI 0–2 μmol/L). Plasma alanine was decreased 100 μmol/L (RI- 131–600 μmol/L) and lysine 140 μmol/L (RI- 84–260 μmol/L). The low alanine concentration in association with elevated branched-chain amino acids is indicative of a true metabolic decompensation.

**Figure 1:**
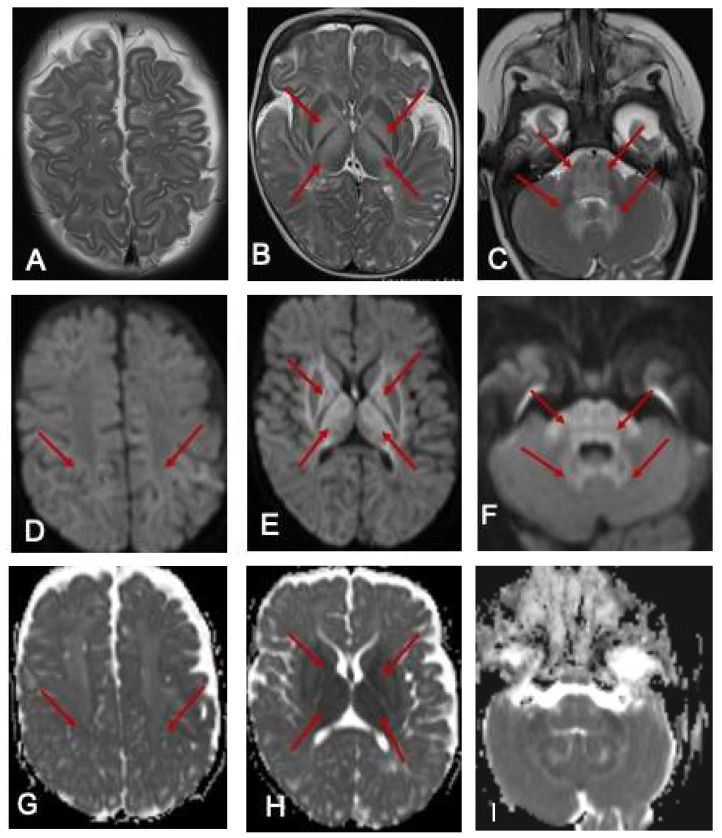
MRI of the brain at 7 months. The red arrows show the hyperintense signal changes. T2-weighted axial images of an MRI scan of the brain at 7 months of age (**A**–**C**) show hyperintense signal changes in the white matter (**A**), globus pallidus (**B**), and thalamus (**B**); pons, cerebellar peduncle, and dentate nucleus (**C** arrows). The corresponding areas show restricted diffusion in DWI Images (**D**–**F**) and ADC maps (**G**–**I**).

Ammonia was 113 μmol/L (10–50 μmol/L). She commenced intravenous dextrose 10% containing fluids and lipids in the acute setting, oral thiamine supplementation, a protein-restricted diet and MSUD-specific, BCAA-free formula (MSUD Anamix Infant, Nutricia Australia). MRI of the brain demonstrated bilateral symmetrical T2 hyperintense signal changes involving the white matter globus pallidus thalamus brainstem and dentate nuclei with restricted diffusion (Figure 1). EEG was mildly abnormal, with intermittent slowing (2–3 Hz amplitude slowing) noted bilaterally and unilaterally in the posterior and frontal regions with occasional generalised bursts and occasional right centrotemporal sharp transients. She responded well to the dietary intervention and was discharged home with residual neurological sequelae including hypotonia and continued involuntary limb movements, which were assessed to be myoclonic jerks.

Molecular analysis of *BCKDHA*, *BCKDHB*, and *DBT* using next-generation sequencing (NGS) identified compound heterozygous variants of uncertain significance (VUS) in the *DBT* gene: c.365A>G; p.Tyr122Cys, and c.434-15_434-4del; p.?. Parental segregation studies confirmed the variants were in trans. The paternal c.434-15_434-4del variant was subsequently reclassified as pathogenic based on a previous report wherein it was detected as compound heterozygous in a patient with MSUD [8]. mRNA analysis revealed that this deletion arose due to an alternative acceptor splice site, which disrupted the open reading frame and may result in a null allele due to nonsense-mediated mRNA decay [10]. Criteria of evidence of pathogenicity considered as per the American College of Medical Genetics and Genomics (ACMG) guidelines were PVS1, PM2, PM3, and PP4-moderate [11]. The maternal c.365A>G variant is reported in the ClinVar database as a VUS (ClinVar Variation ID: 166984; last accessed 28 July 2020). Given that the clinical phenotype was strongly convincing for MSUD, we proceeded to conduct leucine decarboxylation studies in cultured skin fibroblasts as orthogonal biochemical evidence to strengthen the likelihood of pathogenicity of this variant. [1-^14^C]-Leucine decarboxylation studies showed impaired decarboxylation consistent with an intermediate form of MSUD (Table 1). The maternal variant was subsequently reclassified as likely pathogenic based on the ACMG criteria of PM2, PM3, and PP4-moderate.

**Figure 2:**
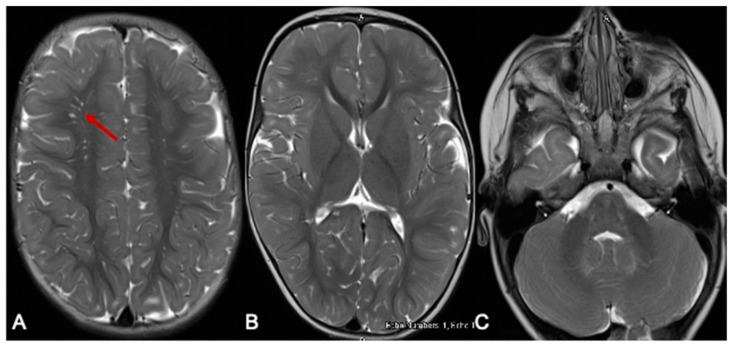
MRI of the brain 10 months later. The red arrow indicates the prominent perivascular spaces. From a follow-up MRI brain scan 10 months later, the T2-weighted axial images show significant improvement in myelination. Prominent perivascular spaces are also seen (**A**-arrow). There is resolution of the previously noted signal changes of basal ganglia and thalamus (**B**), and the brainstem and dentate nuclei (**C**).

Our patient has improved significantly since her diagnosis and is now tracking along her developmental milestones. Formal assessments by her occupational and physiotherapists placed her within age-appropriate milestones at 15 months. Her communication skills were also age-appropriate at 14 months using the Rossetti Infant–Toddler language scale. She has had only one readmission aged 8 months for elevated BCAAs [leucine 859 μmol/L (RI 56–178), isoleucine 392 μmol/L (RI 34–106), valine 643 μmol/L (RI 108–314)], detected on routine dried blood-spot monitoring. She was otherwise clinically well and asymptomatic during this episode. At her last review, aged 2 years, her natural protein intake was 1.16 g/kg/day (14.5 g daily) with a RDI of 1.08/k/day, and BCAA free protein (MSUD Anamix Junior, Nutricia Australia) was 10 g daily. Her weight was just above the 55th percentile and height 75th percentile. Her two- to three-weekly DBS BCAA monitoring levels have remained normal for 8 months, with marginally elevated alloisoleucine (4–26 μmol/L, RI 0–3). Her repeat MRI at 10 months of age showed (Figure 2) complete resolution of the hyperintense signal changes and age-appropriate myelination.

Reclassification of the *DBT* variants has enabled the parents to access prenatal testing for their second pregnancy via chorionic villous sampling and genetic testing.

## 4. Methodology

### 4.1. Plasma Amino Acids Analysis

Samples were deproteinised using s-sulphosalicylic acid precipitation, separated by centrifugation and derivatised with 6-aminoaquinolyl-N-hydroxysuccinimidyl carbamate. Derivatised amino acids were separated using a reverse phase BEH C18 column on a Waters UPLC system (Waters Corporation, MA, USA). The signal was detected by UV absorption at 260 nm and quantified by Empower software (Waters Corporation, Rydalmere, NSW, Australia).

### 4.2. Dried Blood Spot BCAA Analysis

Methanol-containing deuterated internal standards (leucine, isoleucine, and alloisoleucine) was used to measure BCAAs from punched 3 mm dried blood spots. The extract was dried and reconstituted with aqueous mobile phase (H_2_O with 0.1% formic acid and 0.02% heptafluorobutyric acid). A gradient elution using Waters Xevo UPLC system (Waters Corporation, Rydalmere, NSW, Australia) was used to separate the BCAA; with an aqueous mobile phase and organic mobile phase comprising acetonitrile with 0.1% formic acid and 0.02% heptafluorobutyric acid [12].

### 4.3. Leucine Decarboxylation Studies

Cultured skin fibroblasts were harvested at confluence. Cell pellets were resuspended in 0.2 M HEPES buffer containing Earle’s salts and incubated with 1.85 MBq of [1-^14^C] leucine for 1, 2, and 3 h at 37 °C. Duplicate samples were prepared with a control tracer (1.85 MBq of [1-^14^C] ornithine) to monitor sample viability, and heat-inactivated skin fibroblasts were included as reaction blanks. The ^14^CO_2_ released at a given incubation time point was trapped with 1 M KOH and subsequently determined using scintillation counting. The ^14^CO_2_ amount was extrapolated from a calibration curve relating the total radioactivity released against incubation time and expressed per microgram of cell protein.

## 5. Discussion

MSUD is a genotypically and phenotypically heterogeneous disorder of variable clinical severity. Phenotypic distinctions between classic MSUD and partial BCKD enzyme deficiency, which manifests only intermittently or as intermediate or thiamine responsive MSUD are not absolute [1].

The majority of individuals with intermediate MSUD are detected by NBS, although later diagnosis can occur when newborns are not screened for MSUD. These individuals have plasma BCAA concentrations similar to those with the classic MSUD, but with improved leucine tolerance and require less intensive nutritional support during episodes of acute metabolic decompensations [12]. Metabolic intoxication with significant leucinosis and encephalopathy can nevertheless be severe if subjected to sufficient catabolic stress [1].

Prognostication of clinical outcome may be difficult to determine in the acute decompensated state. Dramatic neuroimaging findings in the early stages may not correlate with eventual clinical outcomes [4], as demonstrated in our patient’s significant initial MRI abnormalities that were subsequently resolved with optimal dietary interventions and excellent compliance. This case further substantiates the reversibility of neurological insults sustained during acute decompensation, also reported in other patients where complete resolution of neuroimaging abnormalities has occurred concurrently with normalisation of BCAA levels [13].

Importantly, this report highlights the challenges faced in identifying non-classical forms of MSUD through NBS. A positive NBS result indicative of MSUD in our screening laboratory is defined as a combined leucine and isoleucine level (total leucine) greater than the population 99.9th centile of 400 μmol/L with increased alloisoleucine. False positive NBS results with elevation of BCAAs suggestive of MSUD can occur in newborns receiving total parenteral nutrition. BCAA ratios to phenylalanine or alanine were no longer routinely used at the time of testing this patient as they may not detect patients with milder forms of MSUD, including intermittent or intermediate MSUD [14]. The second tier NBS test for MSUD determines individual BCAAs and includes alloisoleucine in DBS. Elevations would warrant follow-up diagnostic testing in plasma. This approach can aid with reliable determination of classical MSUD cases; however, partial enzyme deficiencies and milder forms, including intermediate MSUD, may not be easily identified [14,15].

During the NBS period, overall findings were found to be inconsistent with classical MSUD and no follow-up plasma samples were requested to pursue diagnostic testing for MSUD due to: (1) patient’s initial NBS with minimally increased “total leucines” (leucine, isoleucine, plus alloisoleucine), (2) second-tier NBS in DBS reported with alloisoleucine level not increased, and (3) the absence of a valine measurement in second-tier DBS analysis, which could help determine the BCAA ratios as supportive evidence.

The subsequent diagnosis of this patient prompted further review of MSUD follow-up procedures, and several service improvements were implemented. These included redetermination of the reference intervals with additional data, addition of valine to second-tier NBS testing and implementation of BCAAs ratios (isoleucine: leucine: valine of 1:2:3) to increase the probability of detection of non-classical MSUD.

Advances in genomic sequencing technologies have resulted in the increasing discovery of novel disease-causing genes or rare variants, with the consequent need for orthogonal functional studies to validate their mutational impact and pathogenicity.

The variants in this 5-month-old girl with biochemical results consistent with MSUD demonstrate the importance of performing functional assays to validate pathogenicity and reclassification of these variants. This is particularly important when future prenatal testing is proposed to be based on molecular analysis alone.

Prior to this study, the reference laboratory had not received requests for leucine oxidation studies for over 7 years. The parents’ desire to expand their family necessitated validation of variant pathogenicity to facilitate prenatal testing. Fortunately, the reference laboratory was confident in their ability to offer the analysis, however it is concerning that the availability of these types of functional studies for some of these rare inborn errors of metabolism has continued to decline. An international collaborative effort may be warranted to preserve the capacity to perform some of these studies, as they are of critical importance in facilitating reclassification of variants of unknown significance. This has significant clinical impact, particularly in prognosticating clinical phenotype and in reproductive counselling. The parents subsequently conceived naturally and proceeded with chorionic villus sampling at which the fetus was determined to be unaffected through genetic analysis of *DBT*.

In summary, we have described a patient with an intermediate form of MSUD who was not initially identified through NBS, because the combined leucine and isoleucine levels were below the laboratory cut-off, and alloisoleucine was determined as not increased. Use of BCAA ratios as part of second-tier and diagnostic testing can improve the detection of non-classical MSUD forms. Leucine oxidation studies have been paramount in verifying the pathogenicity of compound heterozygous VUS and thus enabling reproductive family planning.

## Figures and Tables

**Table 1:** Leucine decarboxylation study results.

Cultured Skin Fibroblast Sample	^14^CO_2_ Released/hour/µg Protein	
	[1-^14^C] Leucine	[1-^14^C] Ornithine
Proband	2.1	31
Mother	28	14
Father	22	16
Normal controls	31, 50	23
MSUD affected	0.29	24
